# Interferon-β inhibits glioma angiogenesis through downregulation of vascular endothelial growth factor and upregulation of interferon inducible protein 10

**DOI:** 10.3892/ijo.2014.2620

**Published:** 2014-08-25

**Authors:** SHINGO TAKANO, EIICHI ISHIKAWA, MASAHIDE MATSUDA, TETSUYA YAMAMOTO, AKIRA MATSUMURA

**Affiliations:** Department of Neurosurgery, Faculty of Medicine, University of Tsukuba, Tsukuba, Ibaraki 305-8575, Japan

**Keywords:** angiogenesis, glioma, interferon-β, interferon inducible protein 10, vascular endothelial growth factor

## Abstract

Interferon-β (IFN-β) has been used clinically for malignant glioma growth inhibition. Recently IFN-β is re-evaluated for its sensitization mechanism to the chemotherapeutic agent temozolomide, because angiogenesis is essential for malignant glioma growth. In this study, we investigated new mechanisms of inhibition of glioma angiogenesis by IFN-β. Three malignant glioma cell lines, U87, TK2 and Becker, were used for *in vitro* study. The effect of IFN-β for these cell lines were evaluated by means of proliferation (MTT assay), conditioned medium induced HUVEC migration, VEGF and interferon inducible protein 10 (IP10, angiogenesis inhibitor) expression by RT-PCR and western blot analysis. SCID mouse U87 subcutaneous model and U87 implant cranial window model were used for *in vivo* study. The effect of IFN-β with the models was evaluated by means of tumor growth, tumor tissue expression for VEGF and IP10, tumor tissue CD31 positive vessel densities, apoptosis and tumor microcirculation (blood velocity, interaction between leukocytes and endothelial cells). *In vitro*, IFN-β upregulated IP10 expression and downregulated VEGF expression time- (4–48 h) and dose- (10–5,000 U/ml) dependently. At the same dose, glioma cell-induced HUVEC migration was inhibited, but cell proliferation was not affected. IFN-β local and systemic injection at 10^5^ U and at 5×10^5^ U/day, for 15 days inhibited U87 subcutaneous growth significantly. In the tumor tissues, VEGF expression and vessel densities were downregulated, but IP10 expression and apoptosis index upregulated. In addition, IFN-β local injection increased collagen fiber deposition in the tumor tissues. IFN-β 5×10^5^ U/day, s.c. injection for 7 days reversed the decreased leukocyte adhesion to endothelial cells, but did not affect blood velocity and vessel images. One of the important roles of IFN-β for malignant glioma growth inhibition was anti-angiogenesis by directly inhibiting angiogenesis through downregulation of VEGF and upregulation of IP-10 and indirectly changing the tumor microcirculation and regulating the interstitial pressure.

## Introduction

Interferons (IFNs) are a family of natural glycoproteins that consist of IFN-α, -β and -γ. The antiviral activity of IFNs led to their discovery, but later data revealed that they also control cell growth and differentiation, inhibit expression of oncogenes, and activate T lymphocytes, natural killer cells and macrophages. Therefore, the efficacy of IFN therapy for various malignancies, including malignant gliomas (1), has been investigated for many years.

Recently anti-glioma action of IFN-β has been re-evaluated. IFN-β markedly enhanced sensitivity to temozolomide (TMZ) via downregulation of MGMT transcription (2,3). The results of the study suggest that compared to TMZ-based chemotherapy plus radiotherapy, chemotherapy with IFN-β and TMZ and concomitant radiotherapy further improve the clinical outcomes of patients with malignant gliomas. A multicenter phase I clinical trial established that therapy with IFN-β and TMZ is safe, well tolerated, and prolongs survival of patients with glioblastoma (4,5). Taken together, IFN-β increased the therapeutic efficiency of TMZ in cases of newly diagnosed primary glioblastoma, particularly in patients with the unmethylated MGMT promoter (6). A prospective randomized control trial to compare the clinical outcomes of newly diagnosed glioblastoma patients treated with TMZ alone or with TMZ and IFN-β combination therapy is ongoing.

Because glioblastoma is one of the most richly neovascularized solid tumors in terms of vasoproliferation, endothelial cell hyperplasia, and endothelial cell cytology (7), antiangiogenic approach may be especially suitable for the treatment of malignant gliomas (8). Recent large clinical study clearly demonstrated the effectiveness of anti-VEGF antibody (bevacizumab) for malignant glioma (9,10). Antiangiogenic activity of IFN-β has been reported previously, IFN-β inhibits some growth factors (bFGF, interleukin 8) (11,12) and gelatinase (13) transcription and/or protein production. In this study, we investigated the antiangiogenic effect of IFN-β for malignant gliomas *in vitro* and *in vivo*, especially about VEGF production, angiogenic stimuli and inhibitor balance, and tumor microcirculation that are not previously proven mechanisms as antiangiogenic actions of IFN-β.

## Materials and methods

### Human glioma cell lines and culture conditions

The human glioma cell line U-87 MG was obtained from the American Type Culture Collection (Rockville, MD). The human glioma cell line TK2 was established from glioblastoma at the Department of Neurosurgery, University of Tsukuba. The human glioma cell line, Becker, was a generous gift. Cells were maintained in MEM supplemented with 10% FCS in a humidified atmosphere containing 5% CO_2_ at 37°C.

Human umbilical cord vein endothelial cells (HUVECs) harvested from umbilical cords were a generous gift of Dr Okuda (University of Tsukuba). HUVECs were maintained with collagen coated flasks (Iwaki Glass, Tokyo, Japan) in E300 medium (Kyokuto, Tokyo, Japan) which are designed for HUVEC culture containing 2% fetal calf serum, heparin, aFGF and EGF.

### Reagents

Human IFN-β was a gift from Toray Industries, Inc. (Tokyo, Japan).

### Cell proliferation assay (MTT assay)

Cell proliferation assays were performed using the CellTiter 96™ Aqueous Non-Radioactive Proliferation Assay (Promega Corp., Madison, WI) as described previously (14). This assay measures the reduction of a tetrazolium compound (3-(4,5-dimethylthiazol-2-yl)-5-(3-carboxymethoxyphenyl)-2-(4-sulfophenyl)-2H-tetrazolium), by living cells to a formazan product. Briefly, the glioma cells, 1×10^5^/ml in DMEM with 10% FCS, were plated in 96-well plates (Becton Dickinston, Lincoln Park, NJ) at 5,000 cells. After 24-h incubation, the various doses of IFN-β (10 to 5,000 U/ml) were added to the wells. The cells were incubated for 48, 96, 144 h. At the end of incubation period, to the microplate wells were added 20 μl of a freshly prepared combined tetrazolium compound and an electron coupling reagent (phenazine methosulfate) solution, then incubated for 2 h at 37°C, and the optical density at 490 nm was read on an automatic microplate reader (Model 550, Bio-Rad). The experiment was repeated at least three times in triplicate wells for each concentration of IFN-β.

### Western blot analysis

The tissues and the cell pellets were homogenized with a ultrasonic homogenizer on ice in 1 ml of extraction buffer [25 mM Tris, 100 mM NaCl, 20 mM NH_4_HCO_3_, pH 7.5, protease inhibitor cocktail; Complete Mini (Roche) one tablet] per 100 mg wet weight of tissue and the protein lysates were obtained after centrifugation at 50,000 × g for 30 min at 4°C. Lysates containing 50 μg of total protein, as estimated by the method of Bradford using bovine serum albumin as a standard, were separated on 12% SDS-polyacrylamide gel, electroblotted onto 0.2 μm nitrocellulose membrane (Bio-Rad) and were immunoassayed with rabbit polyclonal anti-VEGF antibody (A-20, 100 μg/ml, Santa Cruz Biotechnology, Santa Cruz, CA) at the dilution of 1:200 and mouse monoclonal anti-human IP-10 antibody (500 μg/ml, Dako, Glostrup, Denmark) at the dilution of 1:100. The immunocomplexes formed were visualized with alkaline phosphatase-conjugated anti-rabbit or anti-mouse immunoglobulin G (IgG) using ECL western blot analysis system (Amersham Pharmacia Biotech).

### U87 SCID mouse subcutaneous model

After the implantation of 1×10^5^ U87 cells in the flank of 6 weeks old male SCID mouse (Clea Japan), U87 tumor tissue fragments were removed and then re-implanted into another SCID mouse. Harvested tumor fragments, 1 mm^3^ in size, were implanted into the flank of 17 SCDI mice. The animals were divided into 3 groups randomly; 5 control group, 5 IFN-β low dose group, 7 IFN-β high dose group. IFN-β treatment was started at day 7 after the implantation when the subcutaneous tumor reached 5 mm in size. IFN-β was locally injected adjacent to the tumor 1×10^5^ U (low dose group) and 5×10^5^ U (high dose group) once daily for 15 days. Size of the subcutaneous tumor was measured by caliper. At 15 days after the treatment, the tumor tissue was removed. A part of the tissue was immediately fixed in 10% phosphate-buffered formalin for 48 h, paraffin-embedded, and used for routine pathological diagnosis and immunohistochemistry. The other part of the tissue was immediately frozen with liquid nitrogen and stored at −70°C. Two mice of the 7 IFN-β high dose group survived for next 20 days without any treatment and tumor volume was measured.

Similar set of the U87 subcutaneous tumor experiments were repeated with IFN-β systemic (intraperitoneal) injection instead of local injection. Fifteen mice were divided into 3 groups; 5 control, 5 IFN-β low dose and 5 IFN-β high dose. This systemic injection experiment was only used to measure the tumor volume between three groups.

### RNA isolation and reverse transcription polymerase chain reaction (RT-PCR)

Total RNA was extracted from IFN-β treated and control frozen tumor tissues (4 controls, 4 IFN-β low dose, 4 IFN-β high dose) and glioma cell lines treated with IFN-β using RNeasy mini kit (Qiagen GmbH, Germany). Quantitative RT-PCR for IP10 and VEGF mRNA in glioma cells and glioma tissues has been described previously (15). We performed RT-PCR with the GeneAmp™ RNA PCR Kit (Perkin-Elmer Cetus, Norwalk, CT). Briefly, 1 μg of total RNA was reverse transcribed by MuLV reverse transcriptase in the presence of random hexamer, followed by indicated cycles of PCR reaction (95°C for 1 min, 55°C for 1 min and 72°C for 1 min) in the presence of 2 μM IP10 specific primers (32 cycles), VEGF specific primers (28 cycles), or the β-actin specific primers (16 cycles) as a control. The IP10 primers were designed, the reverse primer (5′-GATTCAGACATCTCTTCTCACCC-3′) is complementary to positions 295–275, and the forward primer (5′-TGACTCTAAGTGGCATTCAAGG-3′) corresponds to positions 107–128 (16). The VEGF primers included the reverse primer (5′-CCTGGTGAGAGATCTGGTTC-3′) spanning bases 861-842 and the forward primer (5′-TCGGGCCTCCGA AACCATGA-3′) spanning bases 141–160. The β-actin primers included the reverse primer (5′-GGAGTTGAAGGTAGTTTC GTG-3′) spanning bases 2429-2409 and the forward primer (5′-CGGGAAATCGTGCGTGACAT-3′) spanning bases 2107–2126. The predicted sizes of the amplified IP10 and β-actin DNA products were 188 and 214 bp, respectively. The VEGF primers were chosen because they amplified exons 3 to 8 and allowed for distinguishing between the different VEGF splicing variants. PCR products of 516 and 648 bp corresponded with VEGF121 and VEGF165, respectively. The quantification of these RT-PCR product levels was performed on a Macintosh computer using the public domain NIH Image program (developed at the US National Institute of Health).

### Antibodies and immunohistochemistry

The Dako LSAB Kit for mouse and rabbit primary antibody (Dako) was used. Tissue sections were deparaffined and incubated with 10% normal goat serum in PBS for 20 min. The sections were then incubated with a polyclonal anti-VEGF antibody, A-20 (Santa Cruz Biotechnology) at a dilution of 1/100 (1 μg/ml IgG) in PBS overnight at 4°C, and a monoclonal anti-mouse CD31 antibody (BD Pharmingen) at a dilution of 1/200 in PBS for 60 min at room temperature. Chromatographically purified mouse IgG and rabbit IgG (Dako) at the same IgG concentration were used as negative controls. Sections were incubated with biotin-conjugated goat anti-mouse or anti-rabbit immunogloblin for 10 min, followed by washing in PBS for 10 min. The sections were then incubated with peroxidase conjugated streptavidin solution for 5 min, followed by washing in PBS for 5 min. Sections were then stained with freshly prepared amino-ethylcarbazole solution for 10 min, followed by washing for 5 min in tap water. The sections were then counterstained with hematoxylin and mounted with aqueous mounting media. The intracellular VEGF immunostaining was assessed separately for tumor and endothelial cells using a semiquantitative scale (−, not detected; +, moderate; ++, strong).

### Tumor vascular density

Vascular density was scored using the vasoproliferative component of the MAGS (microscopic angiogenesis grading system) that has been used to quantify angiogenesis in a variety of tumors (17). The number of vessels at 200X field (1.0 mm^2^) was measured in microvessel ‘hot spots’ (i.e., microscopic areas containing the most dense collections of microvessels, as initially identified under low power magnification) with the use of an Olympus microscope, AHBT3 (Olympus, Tokyo, Japan) on CD31 stained tissue sections. Vascular density was defined by averaging the number of vessels in the three most vascularised areas.

### Histochemical detection of apoptotic cells and determination of apoptotic index

Apoptotic cells were visualized using the ApopTag *in situ* detection kit (Oncor, Gaithersburg, MD) as described previously (15). The staining procedures were modified based on the manufacturer’s instructions. Briefly, after deparaffinization and rehydration, the tissues were digested with proteinase K (20 μg/ml in PBS; Wako, Osaka, Japan) for 20 min at room temperature and washed. Slides were then put into 3% H_2_O_2_ for 5 min and washed with PBS. After adding the equilibration buffer for 10 min. TdT enzyme was pipetted onto the sections, which were then incubated at 37°C for 1 h. The reaction was stopped by putting sections in stop/wash buffer. After washing, anti-digoxigenin-peroxidase was added to the slides. Slides were washed, stained with diaminobenzidine (DAKO) substrate, and counterstained with hematoxylin. A specimen known to be positive for apoptotic cells was used as positive control for subsequent staining. Substitution of TdT with distilled water was used as a negative control. The apoptotic index was expressed as the ratio of positively staining tumor cells to all tumor cells, given as a percentage for each case. At least five representative areas without necrosis in a section were selected by light microscopy using 40- to 200-fold magnification. A minimum of 3,000 cells was counted under a 400-fold magnification. Positively staining tumor cells with the morphological characteristics of apoptosis were identified using standard criteria, including chromatin condensation, nucleolar disintegration, and formation of crescentic caps of condensed chromatin at the nuclear periphery.

### Glioma conditioned medium induction of HUVEC migration

Glioma cells (1×10^5^) were plated into a 6-well plate. After incubation for 24 h in MEM with 10% FCS, the medium was changed to MCDB107 with 0.5% FCS containing various concentrations of IFN-β. After 48 h incubation, the conditioned medium was harvested and the concentration of VEGF in glioma conditioned medium was measured using Quantikine™ Human VEGF Immunoassay (R&D Systems, Minneapolis, MN). Endothelial cell migration was evaluated by 24-well modified Boyden chamber (Coster, Cambridge, MA) as described previously. The chamber contains Nucleopore polycarbonate membranes (8-μm pore size) that had been soaked overnight in 0.1% gelatin in 0.1% acetic acid. A total of 100 μl of HUVECs, 2×10^6^ cells/ml in MCDB107 with 0.5% FBS, was plated in upper well and 600 μl of collected conditioned medium was added to lower wells. The assembly was incubated for 6 h. The membrane was removed, fixed in methanol, stained with hematoxylin and the cells in upper surface were gently wiped with cotton swab. The insert was mounted on glass slide. The number of migrated cells was counted from at random five fields using ×25 magnification. Data were expressed as cells per field. One field corresponded to 0.09 mm^2^ (width, 309 μm × height, 291 μm) of the membrane area. The experiment was repeated two times in quadruplicate for each concentration.

### SCID mouse U87 implant cranial window model and quantitation of intravital tumor microcirculation

U87 tumor tissue fragment (1 mm^3^) was implanted on the surface of the SCID mouse cranial window (n=3). IFN-β was injected intraperitoneally for 7 days, and then the cranial window was evaluated for tumor microcirculation. Three series of experimental studies to visualize blood flow dynamics of the tumor microcirculation and to quantify their microhemodynamic parameters were performed (18).

First, by labeling plasma component, the tumor microvasculature was visualized and mapped to obtain information on vascular architecture and dimensions of microvessels. To enhance the contrast of microvessel images against a dark background, a solution of FITC-labeled dextran (FITC-Dx, MW 150,000; Sigma, St. Louis, MO) was intravenously injected (20 mg/ml, 2 ml/kg). This permitted bright fluorescence images of the vascular lumen, and enabled mapping of the vascular architecture and accurate measurements of luminal diameter. The diameter of microvessels was measured carefully with a vernier caliper on the standstill frame of the video-recorded images by playback of a high quality video-cassette recorder (Model BR-S605B). Their average values were calculated from five measurements in each vessel.

Secondary, to visualize the flow behavior of erythrocytes *in vivo* and to measure their velocities, a part of erythrocytes was labeled fluorescently and injected intravenously. The arterial blood (0.2 ml) of a donor mouse was collected from a tail vein into a 1.5 ml test tube containing heparin (100 units) for anticoagulation. The erythrocytes were separated from the plasma by centrifugation and were washed twice with pysiological saline solution. These erythrocytes were then incubated at room temperature with a phosphate-buffered saline (PBS) solution, adjusted to pH 7.8, containing 1 mg/ml fluorescein isothiocyanate (FITC). After 60 min of incubation, the labeled cells were washed twice with a saline solution containing 1% bovine serum albumin to remove uncombined fluorescent dyes. The final volume percent of the labeled cells was adjusted to 50% by adding an isotonic saline solution. These suspensions were injected intravenously through a tail vein. The erythrocyte velocity was calculated by a frame-by-frame analysis and averaged for at least 10 measurements.

Thirdly, to analyze the leukocyte behavior and the leukocyte-endothelium interaction, leukocytes were also labeled fluorscently. A working solution of rhodamine 6G (Sigma) was prepared by dissolving 10 mg of the dye in 40 ml of physiological saline. This solution was diluted with saline until a final concentration of 50 μg/ml. The optimal concetration of the dye for imaging leukocytes was determined from several preliminary experiments. Each solution was freshly prepared on the day of the experiment and filtered through a 0.22 μm filter before each experiment. Leukocytes were found to be visualized by injecting a small bolus of 2 ml/kg of the solution intravenously. Arolling leukocyte was defined as one that marginates along the vessel wall and is clearly dissociated from the bulk of the blood flow. An adhering leukocyte was defined as one that stays stationary during at least 15 sec of the 30-sec observation period (19).

### Statistical analyses

Vascular density, MIB-1 positivity, apoptosis index, tumor volumes, densitometric value of VEGF, IP10 and β-actin, and the parameter of tumor microcirculation (diameter, velocity, number of leukocyte) were expressed as mean ± SD. Statistically significant differences between the groups were determined using a one-way analysis of variance and the Tukey’s test. All p-values are two-sided; values are considered statistically significant for p<0.05.

## Results

### Antiangiogenic activity of IFN-β in vitro

**Figure 1:**
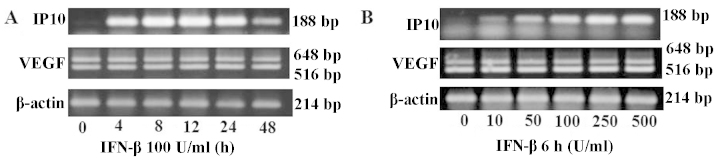
RT-PCR analysis for the transcripts of IP10, VEGF and β-actin in glioma cells, (A) TK2 and (B) Becker. (A) No IP10 mRNA expression in the control, but IFN-β 100 U/ml induced IP10 expression time-dependently between 4- and 48-h treatment. (B) No IP10 mRNA expression in the control, but IFN-β treatment for 6 h induced IP10 expression dose-dependently between 10 and 500 U/ml of IFN-β.

**Figure 2:**
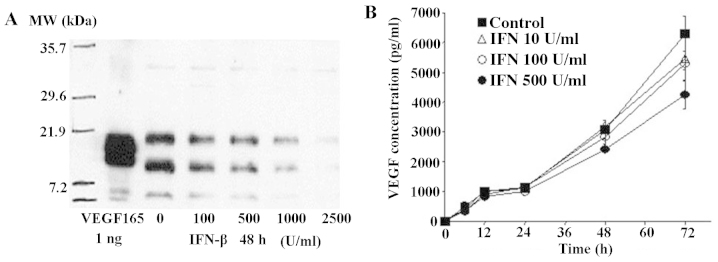
VEGF protein expression of glioma cells. (A) Western blot analysis showed dose-dependent inhibition of Becker secretion of VEGF by IFN-β treatment. (B) VEGF concentration of conditioned medium measured by ELISA was decreased time and dose dependently by IFN-β. The same samples were used for western blot analysis after 50X concentration.

**Figure 3:**
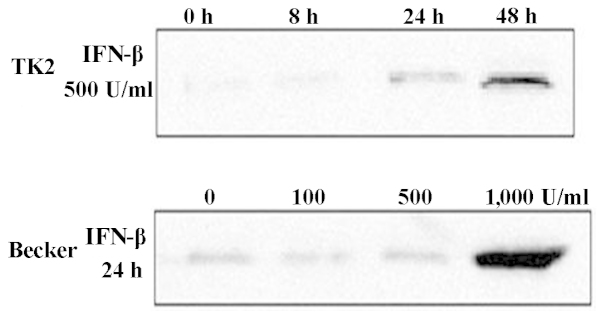
IP10 protein expression of glioma cells. Western blot analysis showed time- (TK2) and dose- (Becker) dependent increase of IP10 expression.

**Figure 4:**
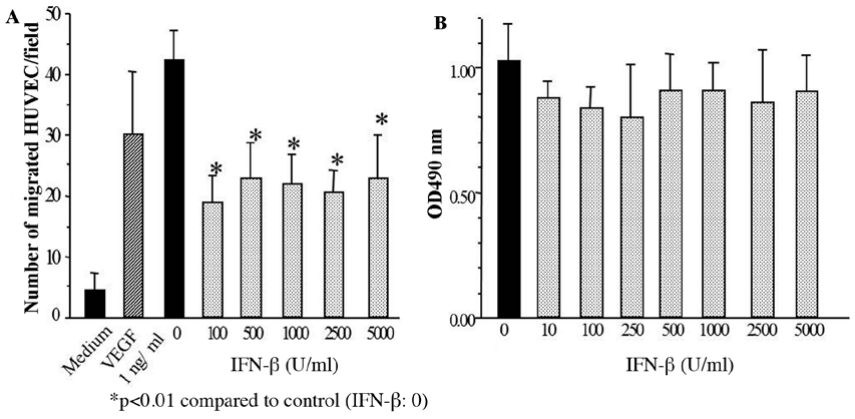
Endothelial cell migration and tumor proliferation. (A) HUVEC migration induced by glioma cell (Becker) conditioned medium. IFN-β at 100 U/ml significantly inhibited HUVEC migration. (B) IFN-β at the dose range 10–5,000 U/ml for 48 h did not inhibit glioma cell (Becker) proliferation measured by MTT assay.

RT-PCR analysis demonstrated IFN-β upregulated IP10 mRNA expression time- (4–48 h) and dose- (10–500 U/ml) dependently, but not VEGF mRNA expression (Fig. 1). However, VEGF protein concentration and secretion in the conditioned medium was decreased time- and dose-dependently by IFN-β treatment (Fig. 2). IP10 protein in cell extracts was increased time- and dose-dependently with IFN-β treatment (Fig. 3). Increased VEGF and decreased IP10 protein expression of glioma cells treated with IFN-β at 100 U/ml for 48 h resulted in the inhibition of HUVECs migration (Fig. 4A). By contrast, glioma cell proliferation was not affected by IFN-β treatment for 48 h at the dose ranging from 10 to 5,000 U/ml (Fig. 4B).

### IFN-β inhibition of glioma growth in subcutaneous tumor

**Figure 5:**
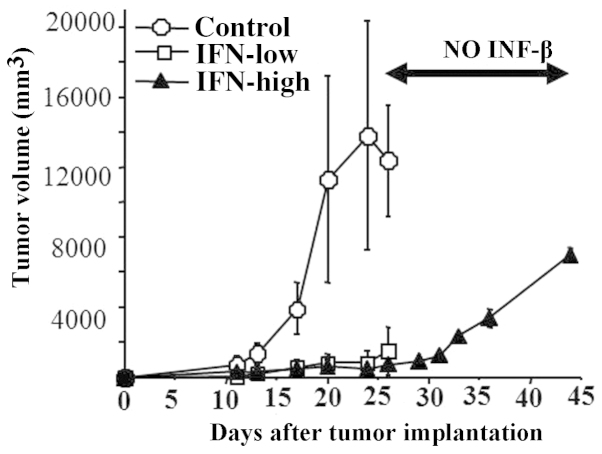
IFN-β inhibition of U87 glioma growth in subcutaneous tumors. Intra tumoral injection of IFN-β low and high dose inhibited U87 subcutaneous tumor growth. Inhibition of tumor growth re-started after discontinuation of IFN-β (after 26th day of tumor implantation).

**Figure 6:**
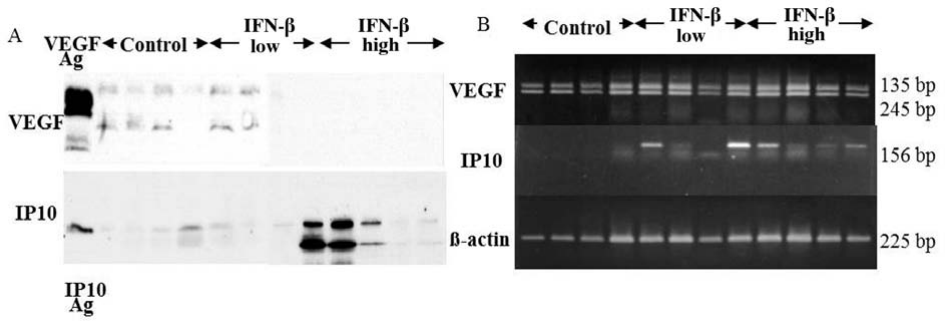
VEGF and IP10 expression in the IFN-β treated and control tumor tissues. (A) Western blot analysis shows IFN-β high dose treatment inhibited VEGF protein expression and induced IP10 protein expression in the tumor tissues. (B) RT-PCR analysis shows IFN-β induction of IP10 mRNA expression in 2 of 4 low dose group and in all of the high dose group.

**Figure 7:**
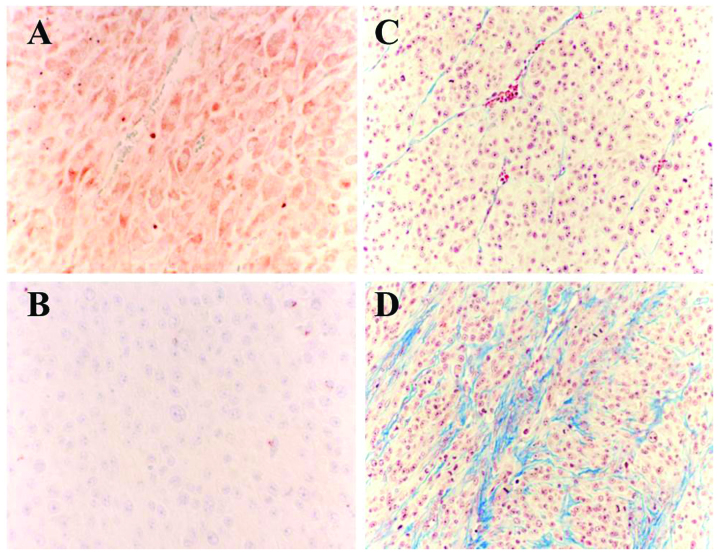
(A and B) VEGF immunohistochemistry and (C and D) Masson’s Trichrome stain of (A and C) control and (B and D) IFN-β high dose group. Note marked decreased expression of VEGF and increased collagen fiber deposition in the treated group. Original magnification, ×100.

IFN-β local injection as well as systemic injection for 15 days significantly inhibited U87 subcutaneous growth (Fig. 5). VEGF protein expression of U87 tumor tissues was decreased dramatically in the IFN-β high dose treatment, while IP10 protein expression increased (Fig. 6A). RT-PCR analysis demonstrated that IP10 mRNA expression of the tumor tissues was not detected in any of control group. Upregulation of IP10 mRNA expression of the tumor tissues was observed in 2 of 4 IFN-β low dose group and all 4 in IFN-β high dose group. VEGF mRNA expression of the tumor tissues was not affected by RT-PCR analysis (Fig. 6B). Immunohistochemistry clearly demonstrated high VEGF expression in the control tumor tissues and decreased VEGF expression in the IFN treated tumor tissues (Fig. 7). VEGF expression was strong in 5 of 5 control group and in none of IFN-β high and low dose group. CD31 positive vessel densities were significantly decreased both in the IFN-β low and high treated groups compared to control group (Table I). In IFN-β high treated group, MIB-1 positivities were significantly low and apoptosis indices were significantly high compared to the control animals (Table I). The growth inhibitory effect of IFN-β was reversible because the tumor did re-start to grow after the discontinuation of the IFN-β treatment at similar degree to control tumors (Fig. 5A). In addition, IFN-β treatment increased collagen fiber deposition in the tumor tissues, which was demonstrated by Masson’s Trichrome stain (Fig. 7C and D). The increase was not observed in the group of systemic IFN-β treatment.

### IFN-β inhibition of glioma microcirculation

With the cranial window model, 7 days after the U87 fragment implantation, tortuous vessels grew around the fragment where red blood cell velocity and the degree of leukocyte adhesion and rolling significantly decreased compared to those of the normal cortical vein at the diameter of 20–80 μm. IFN-β systemic treatment for 7 days dramatically reversed the decreased leukocyte adhesion and rolling in the U87 glioma graft, while tortuous vessel morphology and red blood cell velocity were not unchanged (Table II).

## Discussion

Our study clearly demonstrated that IFN-β inhibited glioma angiogenesis in three different aspects, *in vitro*, subcutaneous tumor, and intracerebral tumor microcirculation. The local and systemic administration of IFN-β to SCID mouse bearing human glioblastoma cells decreased expression of VEGF, induced expression of IP10, reduced vascular density and inhibited reversible tumor growth. Also the systemic administration of IFN-β significantly inhibited glioma growth and reversed the microcirculation of the glioma tissues to that of the non-tumor brain tissue, suggesting the anti-proliferative effect is not due to only high local concentrations of the protein. Among these manifestations, simultaneous action with upregulation of VFGF and downregulation of IP10 is unique. Because angiogenesis is influenced by the balance between stimulatory and inhibitory molecules released by the tumor and its microenvironment, any decrease in a stimulatory molecule or an increase in an inhibitory molecule should reduce the level of neovascularization within the tumor.

### Antiangiogenic action of IFN-β

Clinical data concluded that the systemic chronic administration of IFN-α or IFN-β accelerate the regression of richly vascularized tumors, e.g., life-threatening hemangiomas of infancy (20), hemangio-endotheliomas (21), hemangiopericytoma (22) and Kaposi’s sarcomas (23). The mechanisms responsible for this remarkable clinical outcome remained unclear. IFN-β can inhibit angiogenesis by several mechanisms.

*In vitro*, IFN-β inhibits endothelial cell proliferation (11), endothelial cell migration (24), downregulation of transcription and production of bFGF protein (11), interleukin 8 (25) and collagenase type IV (13), all of which are involved in the angiogenic response. In addition, our data demonstrate IFN-β inhibits production of VEGF protein, although the effects is marginal, and induces production of IP10, endogenous angiogenesis inhibitor, resulting in inhibition of HUVEC migration induced by glioma conditioned medium.

However, the antiangiogenic mechanism of IFN-β for malignant gliomas has not been investigated comprehensively. Boethius *et al* (26) reported that systemic administration of IFN-α to patients with glioblastoma multiforme induced marked changes in the tumor vasculature, which supports the notion that IFN-α may have an effect on tumor vessels. We demonstrated that IFN-β inhibits glioma cell induced endothelial cell migration, VEGF secretion in the glioma cell conditioned medium, and VEGF expression and vessel densities in the glioma tissues. IFN-β is superior to IFN-α in terms of anti-angiogenic effects (27). The above strongly suggests that the *in vivo* antitumor effect of IFN-β in malignant gliomas may be mediated, at least in part, via the angiogenesis inhibition rather than the antiproliferative activity on tumor cells. Hong *et al* (28) demonstrated the level of VEGF and bFGF expression of U87 cells was not influenced by IFN-β treatment at concentrations from 10–500 IU/ml for 24 and 72 h. They measured the expression of cell extract and we measured secreted protein of VEGF.

### IFN-β inhibits VEGF secretion

We reported that IFN-β treatment inhibited glioma cell VEGF secretion *in vitro* and glioma angiogenesis with downregulation of VEGF in glioma tissue. The investigations concerning to relationship of VEGF inhibition and IFN-β are very limited. VEGF promotes phosphorylation-dependent ubiquitination and degradation of IFN receptor and ensuing attenuation of IFN-α/β signaling; these processes appear to be required for efficient angiogenesis (29). In another report, the antitumor effect of IFN-β was offset by the tumor-progressive character of endothelial progenitor cells (EPCs) and the tumor growth, and the vascular density of tumor tissues increased by the co-implanted EPCs were decreased upon IFN-β treatment. In addition, overall expression levels of VEGF in tumor tissues that were decreased upon IFN-β treatment (30).

Recent clinical studies indicate that anti-VEGF agents are important for the treatment of angiogenesis-dependent diseases. The approaches used today are mainly based on the development and administration of functional recombinant protein antagonists that either neutralize the extracellular VEGF function or block VEGF signaling in target cells. The disadvantages of current therapeutic strategies are many, including difficulties in manufacturing active recombinant protein, high-dose requirements, high costs for manufactures and consumers, and the probable need for lifetime treatment of the patient. A novel anti-VEGF strategy by blocking its secretion in tumor cells is reported by retaining a VEGF binding protein in the cell (31), by inhibiting VEGF promoter activity on neuroendocrine tumor cell lines (32), by a PI3 kinase inhibitor in melanoma (33) and by IFN-α in melanoma cell line (34). PI3 kinase activation may occur via loss of phosphatase and tensin homolog (PTEN) that is closely related to IFN-β sensitivity in glioma (35). Taken together, IFN-β treatment appears to be one of novel strategies of anti-angiogenesis on glioma by preventing VEGF secretion.

### IP10 antiangiogenic action

IFN-β upregulates IP10 from a number of cells, including keratinocytes, fibroblasts, endothelial cells, mononuclear phagocytes and cancer cells. IFN-β may shift the biological balance of ELR+ (IL-8) and ELR-CXC (IP10) chemokines, leading to reduced net angiogenic activity (36). IP10 is a potent inhibitor of not only ELR+ CXC chemokine (IL-8) but also the unrelated angiogenic factors bFGF and VEGF (36).

The production of IP10 from adenocarcinoma and squamous carcinoma tumors was inversely correlated with their growth. SCID mouse bearing tumors were given intratumor injection of recombinant human IP10. IP10 treatment resulted in a >40% reduction in tumor size and mass, respectively. The mechanism of growth inhibition by intratumor administration of IP10 was found to be correlated with a reduction in primary tumor-derived angiogenic activity and neovasculature (37). IP10 protein expression was inversely correlated with vascular density and clinical behavior in endometrial cancer (38). We demonstrated IFN-β treatment shifted the balance of VEGF/IP-10 into angiostatic state in glioma cells and tissue. Interestingly, IP-10 has been identified as a major biological marker mediating cancer severity and may be utilized as a prognostic indicator for various cancers (39). IP-10 shows potential as a biological response marker of IFN-β in glioma.

### IFN-β affects tumor microcirculation in gliomas

Yuan *et al* (40) and Foltz *et al* (19) reported the intravital microscopic analysis of malignant gliomas transplanted into a cranial window preparation. Although cranial window model to visualize tumor microcirculation is not a new method, this method is superior to the present MRI based evaluation of tumor vasculature, such as perfusion MRI (41) and vessel architecture imaging (42). Intravital analysis with the cranial window model can provide us information of the tumor microcirculation, such as leukocyte adhesion to vessel wall in addition to vessel architecture. Malignant glioma caused decreased number of leukocyte adhesion to endothelial cells, which is a key factor in the tumor microcirculation. We demonstrated that IFN-β influenced the glioma microcirculation with reversal of the inhibition of leukocyte adhesion. IFN-β inhibits activated leukocyte migration through human brain microvascular endothelial cell monolayer (43). Implantation of IFN-β producing cells upregulated the adhesion molecules, ICAM-1 and VCAM-1 (27). TNF-α by co-treatment with IFN-β increased soluble VCAM-1 in human cerebral endothelial cells (44). VEGF antagonist, such as soluble Flt1 (soluble form of VEGF receptor) increases ICAM1, VCAM1 and leukocyte adhesion in endothelial cells (45). Our data suggest IFN-β increased ICAM1 and VCAM1 directly or indirectly through inhibition of VEGF production with glioma cells, resulting reversal of leukocyte adhesion.

Taken together, the reversal effect of IFN-β on the tumor microcirculation that is considered as a concept for vascular normalization (46), could be one of the mechanisms by which IFN-β treatment exerts antiangiogenic effects in malignant glioma.

### IFN-β affects matrix reaction

Matrix modifications were characterized on histological sections stained with Masson’s Trichrome stain. Collagen deposition or structure of IFN-β treated animals appeared to be thicker than in the non-treated ones. The collagen deposition was prominently observed with local injection of IFN-β, but not with systemic treatment. Combined with the similar increase of collagen fiber in the stromal tissue surrounding the IFN-producing tumor cells (27), high concentration of IFN-β in the tissues is needed for this thicker collagen deposition. Thicker collagen deposition may increase the interstitial pressure in the tumor and decrease diffusion of angiogenic molecules, e.g., VEGF, to the endothelial cells, resulting in inhibition of angiogenesis (47). However IFNs are species specific. Our studies on the effect of the IFNs concerning the host microenvironment (immune, endothelial cells and extracellular matrix) have some limitations.

In conclusion, one of the important roles of IFN-β for malignant glioma growth inhibition was anti-angiogenesis by directly inhibiting angiogenesis through downregulation of VEGF and upregulation of IP-10 and indirectly changing the tumor microcirculation and regulating the interstitial pressure. At present, the clinical effectiveness of IFN-β for human malignant gliomas is limited. Several published studies have shown that anti-angiogenic agents act synergistically with chemotherapy or radiation therapy. Also the growth inhibitory effect was reversible and non-toxic even with high dose of IFN-β in our study, suggesting combination therapy with IFN-β and chemotherapy or radiation therapy for long-term usage will overcome many of the limitations of individual treatment.

## Figures and Tables

**Table I:** U87 tumor pathology 25 days after interferon-β treatment.

	Tumor volume (mm^3^)	MIB-1 (%)	Apoptosis index (%)	Vessel density (no./mm^2^)
Control	12,371.4±3,195.3	38.2±3.9	0.20±0.001	89.0±11.4
IFN-β low	1,523.8±1,354.4^b^	31.4±6.4	0.56±0.104	54.0±11.1^b^
IFN-β high	769.0±339.0^b^	21.8±2.7^a^	1.00±0.024^b^	78.3±8.8^b^

a p<0.05,

b p<0.01 compared to control.

**Table II:** U87 tumor microcirculation with interferon-beta treatment.

	Vessel diameter (μm)	Red blood cell velocity (μm/sec)	Vessel diameter (μm)	Leukocyte adhesion, rolling (no./600 sec)
No tumor	23.9±10.5	475.6±175.3	45.6±34.7	112.1±80.5
U87 tumor	19.8±8.9	248.0±91.2	35.0±21.0	27.9±14.7
U87 tumor, IFN	20.0±0.2	277.2±8.7	66.0±15.2	306.8±131.5^a^

a p<0.05 compared to no treatment.
